# Regulation of inflammatory mediator expression in bovine endometrial cells: effects of lipopolysaccharide, interleukin 1 beta, and tumor necrosis factor alpha

**DOI:** 10.14814/phy2.13676

**Published:** 2018-04-30

**Authors:** Yong Qin Koh, Murray D. Mitchell, Fatema B. Almughlliq, Kanchan Vaswani, Hassendrini N. Peiris

**Affiliations:** ^1^ University of Queensland Centre for Clinical Research Faculty of Medicine The University of Queensland Brisbane Queensland Australia

**Keywords:** Bovine, cytokines, endometrium, epithelial, stromal

## Abstract

An abnormal uterine environment can influence maternal–fetal communication, conception rate and disrupt normal embryo development, thereby affecting fertility and the reproductive performance of dairy cows. Animal variability means that development of endometrial cell lines with appropriate characteristic are required. We evaluated the effect of an infectious agent (i.e., bacterial lipopolysaccharide; LPS) and proinflammatory mediators (i.e., Interleukin 1 beta; IL‐1*β*, and tumor necrosis factor alpha; TNF*α*) on inflammatory mediator gene expression and production by bovine endometrial epithelial (bEEL) and stromal (bCSC) cell lines. Expression of *CXCL8/IL8, IL1A, IL1B*, and *IL6* cytokine genes was significantly upregulated in both epithelial and stromal cells when treated with LPS and IL‐1*β*. LPS treatment of epithelial cells (compared with treatment by IL‐1*β* and TNF*α*) exhibited greater *CXCL8/IL8, IL1A, IL1B, and IL6* cytokine gene expression. Whereas, in stromal cells, IL‐1*β* treatment (compared with LPS and TNF*α*) exhibited greater *CXCL8/IL8, IL1A, IL1B, and IL6* cytokine gene expression. Interestingly, bEEL and bCSC cells treated with IL‐1*β* increased *IL1B* gene expression, suggesting that IL‐1*β* may act unusually in an autocrine‐positive feedback loop. Cytokine production was stimulated by these agents in both cell types. We suggest that the characteristics of these two cell lines make them excellent tools for the study of intrauterine environment.

## Introduction

Bidirectional maternal–fetal communication (i.e., conceptus‐endometrial cross‐talk) is critical for the establishment, maintenance, and progression of a successful pregnancy (Wolf et al. 2003). In cattle embryonic losses that occur in the early days of pregnancy (days 8–17) have been attributed to poor communication between the conceptus and maternal environment (Thatcher et al. 2001). One major factor which can affect the maternal environment and thus the signals between the maternal side and fetal side is the presence of infectious agents in and/or inflammation of the uterus (Groebner et al. 2011). Postpartum uterine disease is one of the leading causes of reduced fertility in dairy cattle. The persistence of pathogenic microorganisms in the uterine lumen following parturition can delay the regeneration of endometrium (Lara et al. 2017; Sheldon et al. 2006) and disrupt the resumption of cyclic ovarian function (Huszenicza et al. 1999; Williams et al. 2008). Significant bacteria responsible for postpartum uterine disease are *Escherichia coli*,* Trueperella pyogenes,* and pathogenic anaerobic bacteria (Sheldon and Owens 2017). The presence of a large number of *Escherichia coli* and their endotoxins, lipopolysaccharides (LPS) are likely to precede and favor the development of uterine infections with other pathogens that disrupt endometrial structure and function in dairy cattle (Dohmen et al. 2000; Magata et al. 2015; Williams et al. 2007). The consequences of uterine bacterial infection, and its associations with clinical and subclinical endometritis can lead to decreased reproductive performance of dairy cattle, compromise animal welfare, and incur economic costs (Carneiro et al. 2016).

Pathogenic microorganisms that invade the female reproductive tract are initially recognized by the innate immune system through the binding of pattern‐recognition receptors (PRRs) to the pathogen‐associated molecular patterns (PAMPs) (Amjadi et al. 2014). PAMPs including LPS; a major constituent of the cell wall unique to most gram‐negative bacteria, strongly activates cells of the immune system and triggers an inflammatory response (Rosenfeld and Shai 2006). The inflammatory intracellular signaling cascades initiated by the interactions between PAMPs and PRRs lead to the production of the primary proinflammatory mediators, including IL‐*β* and TNF*α*, as well as other cytokines and chemokines, which further the activation of complement and the acute phase response to achieve effective immune responses to eliminate the pathogens (Harju et al. 2005; Healy et al. 2014; Splichal and Trebichavsky 2001). However, persistent uterine inflammation due to inadequate pathogen eradication from the uterus, prolonged inflammatory signaling, and defects in anti‐inflammatory mechanisms are likely to be detrimental to fertilization and conception (LeBlanc 2014; Sheldon and Owens 2017).

Most studies (Cronin et al. 2012; Oguejiofor et al. 2015; Swangchan‐Uthai et al. 2012) have focused on the characterization of the inflammatory response triggered by infectious agents. It is also generally considered that postpartum uterine diseases, including clinical and subclinical endometritis, are caused by bacterial infection of the uterus (Foldi et al. 2006). However, the immune system, in particular, the cytokine network is also closely implicated in normal uterine functions including the estrus or menstrual cycle and maintenance of pregnancy (Kelly et al. 2001; Oliveira et al. 2013). Nevertheless, emerging evidence indicates that prolonged proinflammatory events can negatively affect uterine function and impede embryonic development (Hansen et al. 2004; Siemieniuch et al. 2016). The distinction between physiologic and pathologic inflammation of the endometrium, that influences the immune response and regulation, or both remains to be elucidated.

Investigating the methods, processes and signals that are incorporated in maternal–fetal communication are critical in understanding the events that take place during embryo loss/pregnancy failure. Employing animal model systems (especially that of large animals) provides advantages for understanding female reproductive processes (Bahr and Wolf 2012; Cibelli et al. 2013; Swanson and David 2015) but there are limitations and challenges due to the complexities of multicell interactions (Bonney 2013; Renard et al. 2002). We are endeavoring to develop an in vitro model of maternal–fetal communication. This study aims to characterize the maternal cells involved in this model (epithelial and stromal cells from bovine endometrium) to assess their basal and responsive states (to infectious and inflammatory stimuli known to be detrimental to success in pregnancy) and gauge their suitability as cells to be included in our in vitro model.

We therefore evaluated the effect of an infectious agent (e.g., bacterial LPS) and proinflammatory mediators (e.g., IL‐1*β* and TNF*α*) on inflammatory mediator gene expression (e.g., cytokines and chemokines) from bovine endometrial epithelial and stromal cells.

## Materials and Methods

### Endometrial cell lines

A well‐characterized bovine endometrial epithelial (bEEL) and stromal (bCSC) cell lines was utilized for this study (Fortier et al. 1988; Krishnaswamy et al. 2009). bEEL and bCSC cell lines were a generous gift from Professor Michel A. Fortier (Université Laval, Québec). bEEL and bCSC endometrial cells were maintained in 175 cm^2^ (T175, Corning Costar) culture flasks supplemented with exosome‐free media (1640 Roswell Park Memorial Institute (RPMI) medium (Invitrogen, Life Technologies) +10% heat‐inactivated exosome‐free fetal bovine serum (Bovogen, Interpath services Pty Ltd) +1000 U/mL antibiotic‐antimycotic solution [Gibco, Life Technologies]) in a humidified cell culture incubator at 37°C under an atmosphere of 5% CO_2_‐balanced N_2_.

### Endometrial epithelial and stromal cell culture experiments

To evaluate the effects of an infectious agent and inflammatory mediators on the gene expression and cytokine production of endometrial cells, the endometrial epithelial cells (bEEL, seeded at 35 000 cells per well) and stromal (bCSC, seeded at 8000 cells per well) were cultured in a 24‐well culture plates (Corning Costar) supplemented with RPMI media containing bacterial lipopolysaccharide (LPS, 1 *μ*g/mL; O111:B4; catalogue number L2630; Sigma‐Aldrich, St. Louis, MO, USA), bovine IL‐1*β* (10 ng/mL; catalogue number RBOIL1BI; Thermo Scientific; Frederick, MD, USA), bovine TNF*α* (50 ng/mL; catalogue number RPB‐341; IBI Scientific, Peosta, IA, USA) or untreated cells (vehicle control) for 6 h and 24 h. The time of 6 h was chosen based on time‐course studies in endometrium which previously had shown to induces changes in inflammatory genes (e.g., *CXCL8/IL8, IL1B, IL6* and/or fatty acid cyclooxygenase‐2) that peaked by 6 h following the treatment with LPS (Oguejiofor et al. 2015; Swangchan‐Uthai et al. 2012), IL‐1*β* (Huang et al. 1998), and TNF*α* (Arici et al. 1993). While IL6 and IL10 production was assessed using enzyme‐linked immunosorbent assays (ELISA) at 24 h (Healy et al. 2014; Mitchell et al. 2012; Rashidi et al. 2015). Optimal concentrations of LPS, IL‐1*β,* and TNF‐*α* and time‐course for treatments were determined by preliminary experiments (data not shown). The experiment was performed with 4 well replicates per treatment per cell line (*n* = 3). After the treatment, the cells and media were collected and stored at −80°C until further analyses.

### RNA extraction and cDNA synthesis

Total RNA was extracted from the samples (bCSC and bEEL cells) according to Qiagen's manufacturer's protocol using a RNeasy Mini kit (Qiagen, Victoria, Australia) and the contaminating DNA was removed using RNase‐free DNase (Qiagen). The RNA concentration and purity was further determined using a Nanodrop ND‐1000 (Nanodrop Technologies, Wilmington, Delaware). 500 ng of RNA was reverse transcribed into complementary DNA (cDNA) using the RT^2^ First Strand Kit (Qiagen). The cDNA samples were stored at −20°C until further analyses.

### Inflammatory mediator gene expression analyses

RT‐PCR quantification of inflammatory mediator gene expression was performed using the bovine cytokine and chemokine RT^2^ Profiler PCR Array (PABT‐150Z; Qiagen) and the reaction mixture was prepared using the RT^2^ Real‐Timer SyBR Green/ROX PCR Mix kit (Qiagen) following the manufacturer's instructions. Briefly, RT‐PCR was performed using the QuantStudio™ 3 Real‐Time PCR System (Applied Biosystems™, Foster City, California) with an initial 10 min incubation at 95°C followed by 40 cycles at 95°C for 15 sec and 60°C for 60 sec. The specificity of the RT‐PCR products was confirmed by analysis of melting curves. PCR reproducibility, reverse transcription efficiency, and the presence of genomic DNA contamination were verified prior further data analyses using SABiosciences web portal (http://pcrdataanalysis.sabiosciences.com/pcr/arrayanalysis.php). The results for the inflammatory mediator gene expression were normalized against the expression of the endogenous reference genes, glyceraldehyde 3‐phosphate dehydrogenase (*GAPDH*) and TATA box binding protein (*TBP*) which had showed no significant differences in the gene expression between the treatment groups (LPS, IL‐1*β*, and TNF*α*) and untreated (vehicle control) of bCSC and bEEL cells.

### Enzyme‐linked immunosorbent assays

The concentrations of IL6 in the supernatant of cell cultures collected at 24 h were measured using ELISA kits following the manufacturer's instructions (Bovine IL6 ELISA reagent kit ESS0029; Thermo Fisher Scientific; Frederick, Maryland, USA). The IL6 ELISA plate was prepared by coating the wells with 100 *μ*L per well of capture antibody diluted in 0.2 M sodium carbonate‐bicarbonate buffer (pH9.4), sealed with a plate sealer and incubated overnight at room temperature. The coating antibody solutions were aspirated and blocked in assay diluents at room temperature for 1 h. 100 *μ*L of standard or sample was added to the wells and incubated at room temperature for 1 h with moderate shaking. The wells were then washed three times with wash buffer to remove nonspecifically bound materials and added with 100 *μ*L of detecting antibody. After 1 h of incubation at room temperature in 100 *μ*L of detecting antibody, the liquid was aspirated and the wells were washed three times with wash buffer. 100 *μ*L of streptavidin‐HRP diluted in reagent diluent were added to the well and incubated at room temperature for 30 min. Liquid was aspirated and the wells were washed three times with wash buffer to ensure any dried‐on conjugate is resolubilized and washed away. 100 *μ*L of stop solution was added into each well after wells were incubated in the dark for 20 min in 100 *μ*L of substrate solution. The absorbance (absorbance at 450 nm minus absorbance at 550 nm) was measured by the SPECTROstar Nano microplate reader (BMG LABTECH, Offenburg, Germany). The detection range for IL6 is 78–5000 pg/mL.

The concentrations of IL10 in the supernatant of cell cultures collected at 24 h were measured by ELISA kits following the manufacturer's instructions (Bovine IL10 ELISA reagent kit MBS008726; San Diego; California, USA). In brief, 50 *μ*L of standard or sample was added to the wells and incubated at 37°C for 1 h. The wells were then washed four times with wash buffer and added with 50 *μ*L of Chromogen Solution A and 50 *μ*L of Chromogen Solution B to each well successively. The plate was incubated in the dark at 37°C for 15 min and the reaction was stopped with 50 *μ*L stop solution. The absorbance was measured at 450 nm by the SPECTROstar Nano microplate reader (BMG LABTECH). The detection range for IL10 is 15.6–500 pg/mL.

### Statistical analyses

The data analyses for inflammatory mediator gene expression were analyzed through SABiosciences web portal (http://pcrdataanalysis.sabiosciences.com/pcr/arrayanalysis.php) as discussed previously. Statistical analyses were performed using a commercially available software package (Prism 6; GraphPad Inc, La Jolla, CA, USA). Student's *t*‐tests (two‐tailed) were utilized to compare the fold change in inflammatory mediator gene expression between each treatment group (LPS, IL‐1*β*, and TNF*α*), and the vehicle control. The comparison of the IL6 and IL10 production between the treatment groups and the vehicle control were analyzed using a nonparametric Kruskal–Wallis one‐way anova. For all statistical analyses, a *P*‐value of <0.05 was considered statistically significant and any value above it was considered not significant. The results were presented as mean ± SD (*n* = 3 independent experiments).

## Results

### The effect of LPS, IL‐1*β,* and TNF*α* on inflammatory mediator gene expression in bovine endometrial epithelial cells (bEEL)

**Figure 1:**
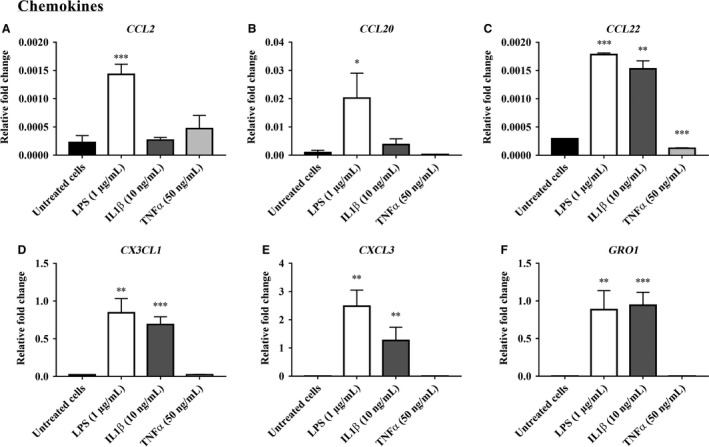
Chemokine gene expression upregulated by LPS, IL‐1*β* and/or TNF*α* in bovine endometrial epithelial cells (bEEL). The relative fold change in chemokine (A) *CCL2*, (B) *CCL20*, (C) *CCL22*, (D) *CX3CL1*, (E) *CXCL3*, and (F) *GRO1* gene expression in bovine endometrial epithelial cells (bEEL) when treated for 6 h with 1 *μ*g/mL LPS, 10 ng/mL IL‐1*β*, and 50 ng/mL TNF*α* compared with untreated bEEL cells (no treatment control). Results are presented as mean ± SD (*n* = 3 independent experiments), and the gene expression were normalized against the expression of the endogenous reference genes (*GAPDH* and *TBP*). Differences in *P < 0.05, **P < 0.01, and ***P < 0.001 were considered statistically significant.

**Figure 2:**
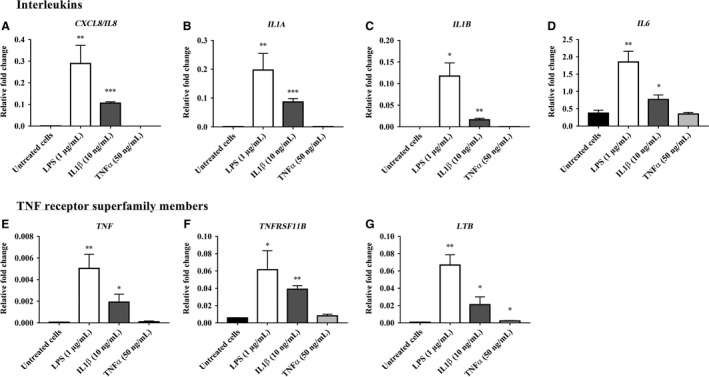
Interleukins and TNF receptor superfamily members’ gene expression upregulated by LPS, IL‐1*β* and/or TNF*α* in bovine endometrial epithelial cells (bEEL). The relative fold change in interleukins (A) *CXCL8/IL8*, (B) *IL1A*, (C) *IL1B*, (D) *IL6*, and TNF receptor superfamily members (E) *TNF*, (F) *TNFRSF11B*, and (G) *LTB* gene expression in bovine endometrial epithelial cells (bEEL) when treated for 6 h with 1 *μ*g/mL LPS, 10 ng/mL IL‐1*β*, and 50 ng/mL TNF*α* compared with untreated bEEL cells (no treatment control). Results are presented as mean ± SD (*n* = 3 independent experiments), and the gene expression were normalized against the expression of the endogenous reference genes (*GAPDH* and *TBP*). Differences in *P < 0.05, **P < 0.01, and ***P < 0.001 were considered statistically significant.

**Figure 3:**
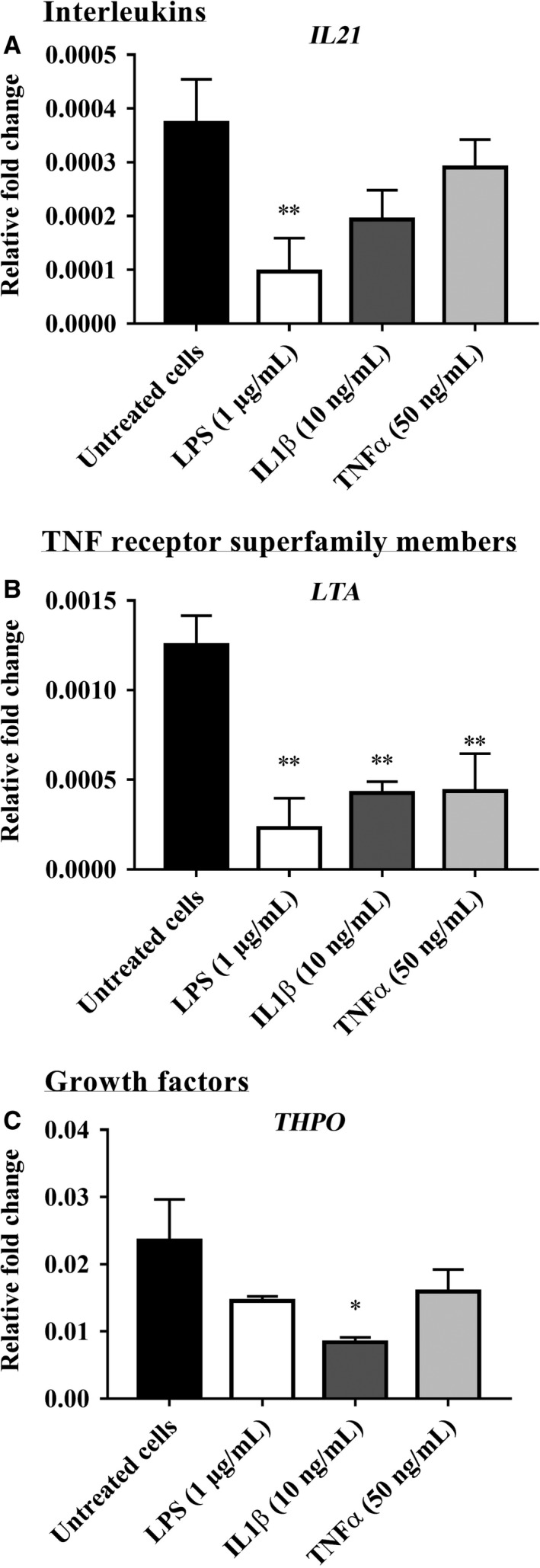
Inflammatory mediator gene expression downregulated by LPS, IL‐1*β,* and TNF*α* in bovine endometrial epithelial cells (bEEL). The gene expression of inflammatory mediator (A) *IL21* (B) *LTA*, and (C) *THPO* when treated with 1 *μ*g/mL LPS, 10 ng/mL IL‐1*β*, and 50 ng/mL TNF*α* for 6 h compared with untreated bEEL cells (no treatment control). Results are presented as mean ± SD (*n* = 3 independent experiments), and the gene expression were normalized against the expression of the endogenous reference genes (*GAPDH* and *TBP*). Differences in *P < 0.05, **P < 0.01, and ***P < 0.001 were considered statistically significant.

The RT‐PCR array data for inflammatory mediator gene expression in bovine endometrial epithelial cells (bEEL) treated with LPS, IL‐1*β*, and TNF*α* are presented in Figures 1, 2, 3 and in Supplemental Data S1. From a total of 84 genes analyzed, 16 inflammatory mediator genes; which met the cycle threshold cut‐offs (<35) for the gene expression data, were significantly altered in bEEL cells by LPS, IL‐1*β* and/or TNF*α*. We present the gene expression that was not determined by RT‐PCR or was not altered by LPS, IL‐1*β* and/or TNF*α* treatment on bEEL cells in Supplemental Data S1. The data presented for the inflammatory mediator gene expression by RT‐PCR are categorized into groups that showed upregulated chemokines (Fig. 1), upregulated interleukins and TNF receptor superfamily members (Fig. 2), and downregulated gene expression of inflammatory mediators (Fig. 3) following treatment with LPS, IL‐1*β* and/or TNF*α* treatment on bEEL cells. bEEL cells challenged by LPS and IL‐1*β* induced a proinflammatory response by upregulating the expression of chemokines; *CCL22, CX3CL1, CXCL3,* and *GRO1* (Fig. 1), interleukins; *CXCL8/IL8, IL1A, IL1B,* and *IL6*, and the TNF receptor superfamily members; *TNF*,* TNFRSF11B*, and *LTB* (Fig. 2). However, the gene expression of *IL21, LTA,* and *THPO* expression was downregulated (Fig. 3). LPS (1 *μ*g/mL) treated bEEL cells (Figs. 1, 2) were shown to induce greater fold change in the gene expression of chemokines (*CCL2, CCL20, CCL22, CX3CL1,* and *CXCL3*), interleukins (*CXCL8/IL8, IL1A, IL1B,* and *IL6*), and TNF receptor superfamily members (*TNF, TNFRSF11B,* and *LTB*), when compared with IL‐1*β* (10 ng/mL) and untreated bEEL cells. In comparison with untreated bEEL cells, bEEL cells stimulated with 50 ng/mL TNF*α* (Figs. 1, 2, 3) did not significantly alter the expression of inflammatory mediator genes.

### The effect of LPS, IL‐1*β,* and TNF*α* on inflammatory mediator gene expression in bovine endometrial stromal cells (bCSC)

**Figure 4:**
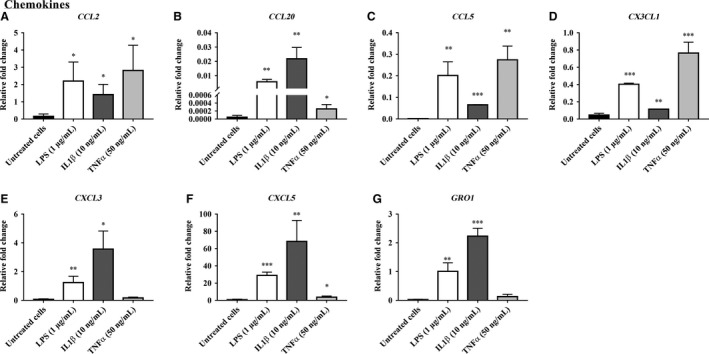
Chemokine gene expression upregulated by LPS, IL‐1*β* and/or TNF*α* in bovine endometrial stromal cells (bCSC). The relative fold change in chemokine (A) *CCL2*, (B) *CCL20*, (C) *CCL5*, (D) *CX3CL1*, (E) *CXCL3*, (F) *CXCL5*, and (G) *GRO1* gene expression in bovine endometrial stromal cells (bCSC) when treated for 6 h with 1 *μ*g/mL LPS, 10 ng/mL IL‐1*β*, and 50 ng/mL TNF*α* compared with untreated bCSC cells (no treatment control). Results are presented as mean ± SD (*n* = 3 independent experiments), and the gene expression were normalized against the expression of the endogenous reference genes (*GAPDH* and *TBP*). Differences in *P < 0.05, **P < 0.01, and ***P < 0.001 were considered statistically significant.

**Figure 5:**
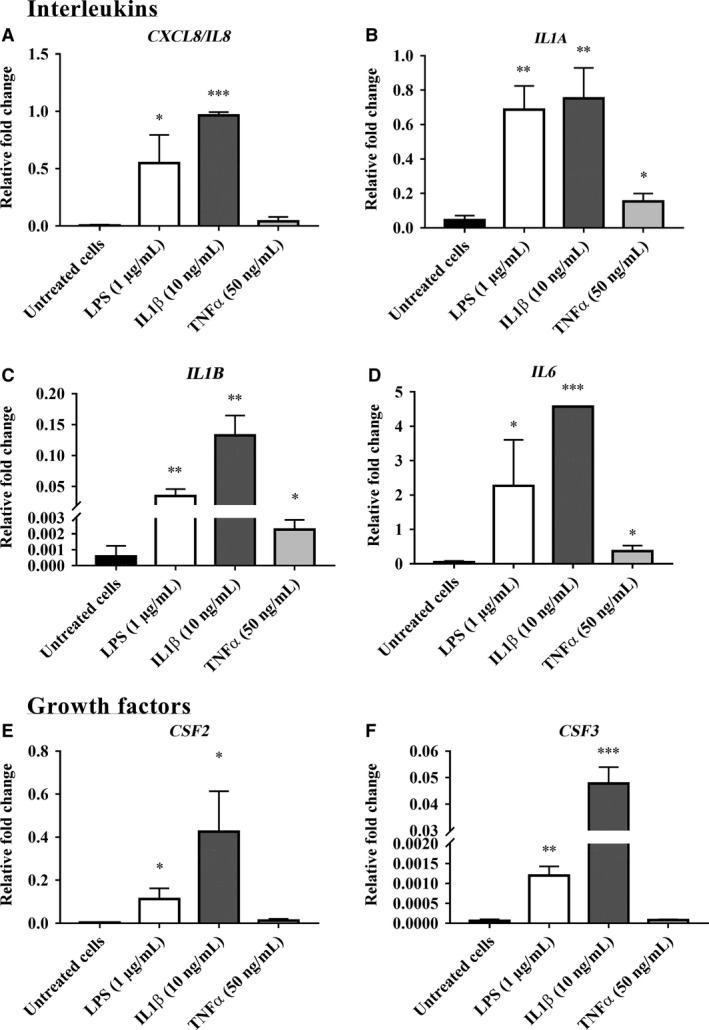
Interleukins and growth factors’ gene expression upregulated by LPS, IL‐1*β* and/or TNF*α* in bovine endometrial stromal cells (bCSC). The relative fold change in interleukins (A) *CXCL8/IL8*, (B) *IL1A*, (C) *IL1B*, and (D) *IL6*, and growth factors (E) *CSF2*, and (F) *CSF3* gene expression in bovine endometrial stromal cells (bCSC) when treated for 6 h with 1 *μ*g/mL LPS, 10 ng/mL IL‐1*β*, and 50 ng/mL TNF*α* compared with untreated bCSC cells (no treatment control). Results are presented as mean ± SD (*n* = 3 independent experiments), and the gene expression were normalized against the expression of the endogenous reference genes (*GAPDH* and *TBP*). Differences in *P < 0.05, **P < 0.01, and ***P < 0.001 were considered statistically significant.

**Figure 6:**
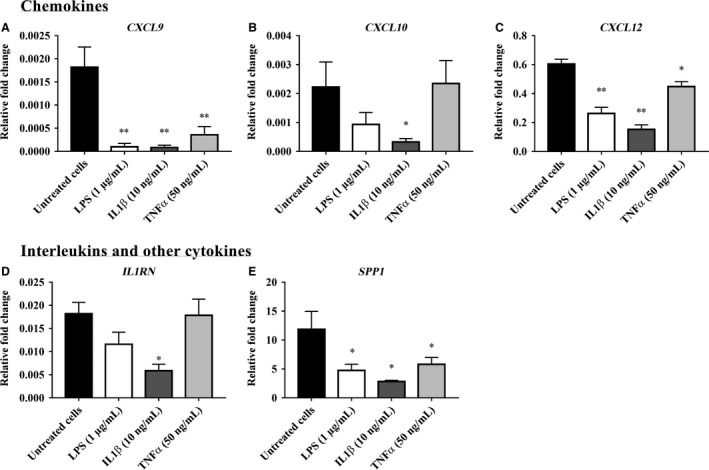
Inflammatory mediator gene expression downregulated by LPS, IL‐1*β* and TNF*α* on bovine endometrial stromal cells (bCSC). The gene expression of inflammatory mediator (A) *CXCL9*, (B) *CXCL10*, (C) *CXCL12*, (D) *IL1RN*, and (E) *SPP1* when treated with 1 *μ*g/mL LPS, 10 ng/mL IL‐1*β*, and 50 ng/mL TNF*α* for 6 h compared with untreated bCSC cells (no treatment control). Results are presented as mean ± SD (*n* = 3 independent experiments), and the gene expression were normalized against the expression of the endogenous reference genes (*GAPDH* and *TBP*). Differences in *P < 0.05, **P < 0.01, and ***P < 0.001 were considered statistically significant.

The RT‐PCR array data for inflammatory mediator gene expression in bovine endometrial stromal cells (bCSC) treated with LPS, IL‐1*β*, and TNF*α* are presented in Figures 4, 5, 6 and in Supplemental Data S2. From a total of 84 genes analyzed, 18 inflammatory mediator genes; which met the cycle threshold cut‐offs (<35) for the gene expression data, were significantly altered in bCSC cells by LPS, IL‐1*β* and/or TNF*α*. We present the gene expression that was not determined by RT‐PCR or was not altered by LPS, IL‐1*β* and/or TNF*α* treatment on bCSC cells in Supplemental Data S2. The data presented for the inflammatory mediator gene expression by RT‐PCR are categorized into groups that showed upregulated chemokines (Fig. 4), upregulated interleukins and growth factors (Fig. 5), and downregulated gene expression of inflammatory mediators (Fig. 6) following treatment with LPS, IL‐1*β* and/or TNF*α* treatment on bCSC cells. bCSC cells challenged by LPS, IL‐1*β*, and TNF*α* (Figs. 4, 5, 6) significantly upregulated the expression of chemokines; *CCL2, CCL20, CCL5, CX3CL1,* and *CXCL5* (Fig. 4) and interleukins; *IL1A, IL1B*, and *IL6* (Fig. 5), and downregulated *CXCL9, CXCL12,* and *SPP1* expression (Fig. 6). The treatment with 10 ng/mL IL‐1*β* onto bCSC cells (Figs. 4, 5) showed greater fold change in the gene expression of chemokines (*CCL20, CXCL3, CXCL5,* and *GRO1*), interleukins (*CXCL8/IL8, IL1B*, and *IL6*), and growth factors (*CSF2* and *CSF3*), when compared with untreated bCSC cells and treatment by 1 *μ*g/mL LPS (Figs. 4, 5). bCSC cells stimulated with 50 ng/mL TNF*α* (Fig. 4) only showed greater fold change in *CCL2, CCL5,* and *CX3CL1* chemokine gene expression when compared with untreated bCSC cells and treatment by 1 *μ*g/mL LPS and 10 ng/mL IL‐1*β*.

### The effects of LPS, IL‐1*β,* and TNF*α* on IL6 and IL10 production by bovine endometrial epithelial (bEEL) and stromal (bCSC) cells

**Figure 7:**
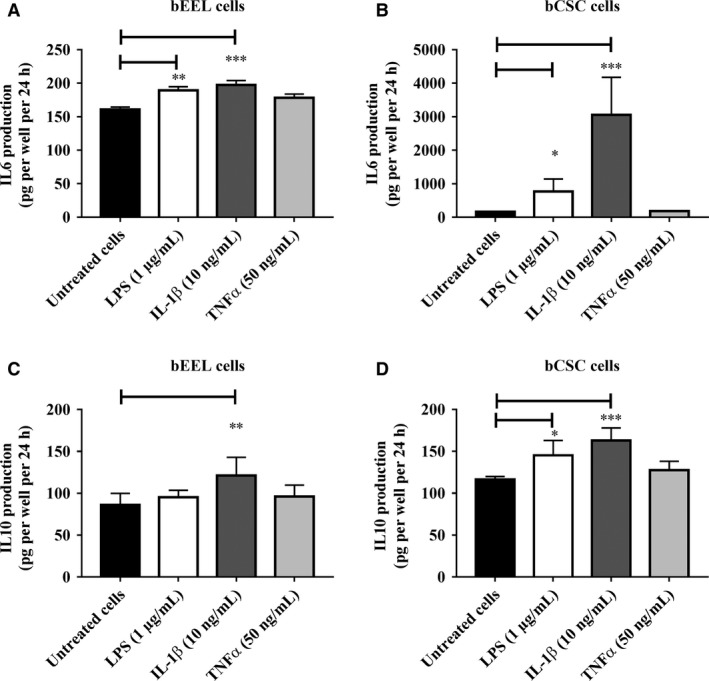
The effect of LPS, IL‐1*β,* and TNF*α* on IL6 and IL10 production by bovine endometrial epithelial (bEEL) and stromal (bCSC) cells. Bovine endometrial epithelial (bEEL) and stromal (bCSC) cells were treated with 1 *μ*g/mL LPS, 10 ng/mL IL‐1*β*, and 50 ng/mL TNF*α* for 24 h. IL6 and IL10 production was determined using ELISAs. (A) IL6 production by bEEL cells treated with LPS, IL‐1*β*, and TNF*α*. (B) IL6 production by bCSC cells treated with LPS, IL‐1*β*, and TNF*α*. (C) IL10 production by bEEL cells treated with LPS, IL‐1*β*, and TNF*α*. (D) IL10 production by bCSC cells treated with LPS, IL‐1*β*, and TNF*α*. Results are presented as mean ± SD (*n* = 3 independent experiments). Differences in *P < 0.05, **P < 0.01, and ***P < 0.001 were considered statistically significant.

The changes in IL6 and IL10 cytokine production elicited by LPS, IL‐1*β*, and TNF*α* on bovine endometrial epithelial (bEEL) and stromal (bCSC) cells are presented in Figure 7. IL6 production was significantly higher when bEEL (Fig. 7A) and bCSC cells (Fig. 7B) were treated with 1 *μ*g/mL LPS, and with 10 ng/mL IL‐1*β* when compared with untreated cells (vehicle control). The anti‐inflammatory IL10 production was only significantly increased when bEEL cells were treated with 10 ng/mL IL‐1*β* (Fig. 7C). bCSC cell production of IL10 was significantly greater when bCSC cells (Fig. 7D) were treated with 1 *μ*g/mL LPS, and with 10 ng/mL IL‐1*β* when compared with untreated cells (vehicle control). No significant differences in the production of IL6 and IL10 were observed when bEEL and bCSC cells were treated with 50 ng/mL TNF*α* when compared with untreated cells (Fig. 7A–D).

## Discussion

Bovine endometrial epithelial and stromal cells challenged by LPS in our study showed higher expression levels of proinflammatory interleukins (*CXCL8/IL8, IL1A, IL1B,* and *IL6*) and chemokines (*CCL2, CCL20, CCL22, CCL5, CX3CL1, CXCL3, CXCL5* and *GRO1*) than untreated cells. Treatment with LPS also induced the production of inflammatory mediator IL6 above basal levels. Our findings are consistent with the published literature involving the upregulation of proinflammatory interleukins including CXCL8/IL8, IL‐1*α*, IL‐1*β*, and IL6 in the pathogenesis of bacterial infection (Herath et al. 2009; Modat et al. 1990; Turner et al. 2014); an example of which is the attraction of neutrophils in response to increased CXCL8/IL8 production which can lead to the formation of pus in the uterine lumen (Haas et al. 2016). Also reported are key roles for IL‐1*α*, IL‐1*β*, and IL6 in the stimulation of acute phase responses as seen in animals with postpartum uterine diseases (Brodzki et al. 2015; Fumuso et al. 2003; Herath et al. 2009). Moreover, the elevated mRNA expression of chemokines *CCL5* (Arima et al. 2000), *CXCL5* (Karlsson et al. 2015), and *GRO1* (Oral et al. 1996) have been reported to have a role in the pathogenesis of endometriosis in human and canine (Karlsson et al. 2015).

Many studies have shown that LPS promotes T helper 1 (Th1‐type) responses and can also bias the immune responses toward the differentiation of other T helper lineages (McAleer and Vella 2008; Shi et al. 2013). Upregulated interleukin (*CXCL8/IL8, IL1A, IL1B,* and *IL6*) and chemokine (*CCL20* and *CXCL5*) have also been reported toward favoring T helper 17 (Th17) responses (Acosta‐Rodriguez et al. 2007; Chung et al. 2009; Disteldorf et al. 2015; Gasch et al. 2014; Hirota et al. 2007; Kimura et al. 2007). The responses to LPS identified in our study by bovine endometrial epithelial and stromal cells suggests that these cells may also favor T helper 17 (Th17) responses and if given the opportunity could promote neutrophilic infiltration to eliminate pathogens.

Pathogens that are not eradicated by the host immune response may persist in the uterine lumen and can trigger aberrant expressions of proinflammatory cytokines which are associated with greater inflammation. In our study the treatment of bovine endometrial and stromal cells with proinflammatory mediatory IL‐1*β* induced the gene expression of proinflammatory interleukins and chemokines when compared with untreated cells. The genes with upregulated expression differed between each cell type. Epithelial cells showed an upregulation in genes associated with TNF receptor superfamily members, however, stromal cells did not show any difference in these genes. Stromal cells did, however, show higher expression of growth factors (*CSF2* and *CSF3*) which were not identified in epithelial cells. Endometrial epithelial cells exposed to IL‐1*β* induced the secretion of the anti‐inflammatory IL10 a response not seen when these cells were exposed to LPS. This suggests that the secretion of IL10, by epithelial cells may help to suppress the activated proinflammatory response. In contrast, endometrial stromal cells exposed to IL‐1*β* increased the production of both IL‐6 and IL‐10. This differential gene expression and cytokine production may be indicative of the responses evidenced in communications between the cells (Goffin et al. 2002) to support individual cellular response, that is stromal cells in supporting epithelial cell functions to respond in a controlled manner as the first line of defense against infectious agents and/or inflammatory mediators (Chen et al. 2013; Srivastava et al. 2013).

The differences in the inflammatory mediator expression between bovine endometrial epithelial and stromal cells responding to IL‐1*β* stimulation may be associated with their specific physiological functions during estrus or menstrual cycle and pregnancy (Geisert et al. 2012; Ross et al. 2003; Sykes et al. 2012). The upregulation of these interleukins (*CXCL8/IL8* – Arici et al. 1993; *IL1B*, and *IL6* – Chalpe et al. 2015), chemokines (*CCL20, CXCL3, CXCL5* – Rossi et al. 2005, and *GRO1* – Nasu et al. 2001), and growth factors (*CSF2* and *CSF3*; Chegini et al. 1999; Rossi et al. 2005) by IL‐1*β* has also been observed and reported previously by others, suggesting that IL‐1*β* can have multiple roles in endometrial physiology including the modulation of endometrial prostaglandin secretion (Davidson et al. 1995; Seo et al. 2012). Interestingly, the upregulation of IL‐1*β* expression in both endometrial stromal and epithelial cells treated with IL‐1*β* implies that IL‐1*β* can act in an autocrine and paracrine manner and plays an important role in mammalian endometrial physiology (Tanaka et al. 2000).

The treatment of both the bovine endometrial epithelial and stromal cells with TNF*α* in our study resulted in a lower stimulatory response than treatment with LPS and IL‐1*β* as the gene expression of many cytokines and chemokines and the secretion of proinflammatory IL6 and anti‐inflammatory IL10 were unchanged. Similar finding was also reported by Chalpe et al. (2015) that telomerase‐immortalized human endometrial stromal cells (T‐HESC) treated with TNF*α* did not enhance the gene expression of CXCL8/IL8, IL‐1*β*, and IL6. TNF*α* did, however, significantly induce chemokines *CCL2, CCL20, CCL5, CX3CL1,* and *CXCL5* expression in bovine endometrial stromal cells, similar to the results of a study by Liu et al. (2011), suggesting that TNF*α* can induce cells to release chemokines to direct leukocytes migration to the site of inflammation, which is often associated with chronic inflammation and cancerogenesis (Zelova and Hosek 2013).

In conclusion, our findings show that epithelial and stromal cells of the endometrium have roles in immunity and can elicit an immune response to bacterial LPS and the proinflammatory mediators (IL‐1*β*, and TNF*α*). Although these events have been described in the endometrium of dairy cattle associated with postpartum uterine disease (Kasimanickam et al. 2013), the distinction between physiologic and pathologic inflammation of the endometrium that influences the immune response and regulation, or both remains controversial. Nevertheless, chemokines and cytokines are key modulators of inflammation and can be classified based on the nature of the immune response. The responsiveness of the cells reassures us of their suitability as the maternal cell component of the in vitro model of maternal–fetal communication that we endeavor to develop and utilize in our future studies. In subsequent studies utilizing these cells in the evaluation of maternal–fetal communication further mechanistic investigations should be included. Manipulating the response of these cells by, for example, with antagonists that block the signaling pathways involved in immune modulation (e.g., TLR4 inhibitors) and the use of treatments against inflammation (e.g., nonsteroidal anti‐inflammatory drugs) will provide further information that may uncover the mechanism(s) at play or help in the development of potential therapies to reverse the disadvantageous effects of infection and inflammation in maternal–fetal communication.

## Conflict of Interest

None declared.

## Data Accessibility

## Supporting information




**Data S1.** bEEL Gene expression (2^−ΔΔCt^).

**Data S2.** bCSC Gene expression (2^−ΔΔCt^).
