# Supplementary material for: Regulation of inflammatory mediator expression in bovine endometrial cells: effects of lipopolysaccharide, interleukin 1 beta, and tumor necrosis factor alpha
Source: Physiol Rep. 2018 Apr 30;6(9):e13676. doi: 10.14814/phy2.13676 (PMC5925570; doi:10.14814/phy2.13676)
Supplement: Supplementary file 1 — Data S1. bEEL Gene expression (2−ΔΔCt). [file PHY2-6-e13676-s001.pdf]

bEEL Gene Expression (2^-ΔΔCt)

	Control		LPS		IL-1β		TNFα	
	Mean	SD	Mean	SD	Mean	SD	Mean	SD
ADIPOQ	0.000145475	2.32029E-05	0.000359565	0.000307515	0.000317233	0.000224979	UNDETERMINED	UNDETERMINED
BMP2	0.475720277	0.055580672	0.457467016	0.043483566	0.219912674	0.083530405	0.418065304	0.042751765
BMP3	0.050962089	0.008343653	0.104221271	0.043178715	0.071664859	0.0061278	0.062854281	0.031099047
BMP6	UNDETERMINED	UNDETERMINED	UNDETERMINED	UNDETERMINED	UNDETERMINED	UNDETERMINED	UNDETERMINED	UNDETERMINED
BMP7	0.650433071	0.137013244	0.659763423	0.305387491	0.569453864	0.023375837	0.657916256	0.266831609
C5	UNDETERMINED	UNDETERMINED	UNDETERMINED	UNDETERMINED	UNDETERMINED	UNDETERMINED	UNDETERMINED	UNDETERMINED
CCL1	0.000300007	0.000149367	0.000229184	8.92931E-05	0.000109798	4.76201E-05	0.000175469	3.69462E-05
CCL11	UNDETERMINED	UNDETERMINED	UNDETERMINED	UNDETERMINED	UNDETERMINED	UNDETERMINED	UNDETERMINED	UNDETERMINED
CCL17	0.731314081	0.204119654	0.65996603	0.180960247	0.329294173	0.17323925	0.526117125	0.196226472
CCL19	UNDETERMINED	UNDETERMINED	UNDETERMINED	UNDETERMINED	UNDETERMINED	UNDETERMINED	UNDETERMINED	UNDETERMINED
CCL2	0.000224618	0.00012261	0.001430215	0.00018065	0.000267627	7.94957E-05	0.000470725	0.000233072
CCL20	0.000949851	0.000800839	0.020226257	0.008804782	0.003750102	0.002080348	0.000286865	1.11652E-05
CCL21	UNDETERMINED	UNDETERMINED	UNDETERMINED	UNDETERMINED	UNDETERMINED	UNDETERMINED	UNDETERMINED	UNDETERMINED
CCL22	0.000292194	UNDETERMINED	0.001782735	2.83474E-05	0.001529883	0.000140875	0.000124249	8.79564E-06
CCL24	UNDETERMINED	UNDETERMINED	UNDETERMINED	UNDETERMINED	UNDETERMINED	UNDETERMINED	UNDETERMINED	UNDETERMINED
CCL3	5.36184E-05	2.90429E-05	UNDETERMINED	UNDETERMINED	1.99544E-05	1.00598E-05	9.52803E-05	5.78432E-05
CCL4	UNDETERMINED	UNDETERMINED	0.000217309	0.000189288	UNDETERMINED	UNDETERMINED	UNDETERMINED	UNDETERMINED
CCL5	0.002313906	0.001985987	0.002258663	0.000653163	0.001656993	0.000669332	0.001419363	0.000578861
CCL8	UNDETERMINED	UNDETERMINED	UNDETERMINED	UNDETERMINED	UNDETERMINED	UNDETERMINED	UNDETERMINED	UNDETERMINED
CD40LG	UNDETERMINED	UNDETERMINED	UNDETERMINED	UNDETERMINED	UNDETERMINED	UNDETERMINED	UNDETERMINED	UNDETERMINED
CD70	0.001100936	0.000728655	0.001896826	0.001269106	0.00130442	0.000727764	0.001161076	0.000606553
CNTF	0.001577205	0.000375381	0.001712357	0.000561836	0.001185143	0.000403581	0.001848036	0.001012094
CSF1	0.018420969	0.002946146	0.048315702	0.034055588	0.036443209	0.021522242	0.029469635	0.013583993
CSF2	0.587067527	0.044868589	1.609367097	0.116232203	0.735719135	0.322563074	0.597381298	0.110895541
CSF3	0.000173287	0.000265823	6.69428E-05	1.1506E-05	7.25028E-05	5.72861E-05	0.000121099	8.24448E-05
CTF1	0.01177656	0.002745952	0.013869797	0.007149738	0.013070912	0.002791189	0.012486385	0.003444394
CX3CL1	0.021526013	0.002408761	0.844603951	0.187928003	0.68911271	0.102378728	0.02265034	0.004980852
CXCL10	UNDETERMINED	UNDETERMINED	UNDETERMINED	UNDETERMINED	UNDETERMINED	UNDETERMINED	UNDETERMINED	UNDETERMINED
CXCL11	UNDETERMINED	UNDETERMINED	UNDETERMINED	UNDETERMINED	UNDETERMINED	UNDETERMINED	UNDETERMINED	UNDETERMINED
CXCL12	UNDETERMINED	UNDETERMINED	UNDETERMINED	UNDETERMINED	UNDETERMINED	UNDETERMINED	UNDETERMINED	UNDETERMINED
CXCL13	UNDETERMINED	UNDETERMINED	UNDETERMINED	UNDETERMINED	UNDETERMINED	UNDETERMINED	UNDETERMINED	UNDETERMINED
CXCL16	0.86206425	0.141020072	1.741644379	0.370142218	1.11829701	0.332025451	0.933905707	0.208784275
CXCL3	0.003895541	0.001180599	2.482796934	0.569318623	1.264546181	0.472045194	0.004121807	0.001372479
CXCL5	0.032951286	0.005020122	16.14554585	3.39699853	13.56995651	4.203791241	0.044171568	0.003666788
CXCL8	0.000339387	0.000268384	0.289314905	0.083747818	0.106282015	0.006257389	0.000232166	5.2953E-05
CXCL9	UNDETERMINED	UNDETERMINED	UNDETERMINED	UNDETERMINED	UNDETERMINED	UNDETERMINED	UNDETERMINED	UNDETERMINED
FASLG	UNDETERMINED	UNDETERMINED	UNDETERMINED	UNDETERMINED	UNDETERMINED	UNDETERMINED	UNDETERMINED	UNDETERMINED
GPI	0.711616383	0.090201667	0.961190003	0.545147795	1.011511111	0.282836667	0.761482151	0.165227994
GRO1	0.002795869	0.00103163	0.884426683	0.252853318	0.943161342	0.170743403	0.002674761	0.001640044
IFNG	UNDETERMINED	UNDETERMINED	UNDETERMINED	UNDETERMINED	UNDETERMINED	UNDETERMINED	UNDETERMINED	UNDETERMINED
IL10	UNDETERMINED	UNDETERMINED	UNDETERMINED	UNDETERMINED	UNDETERMINED	UNDETERMINED	UNDETERMINED	UNDETERMINED
IL12A	0.000313697	0.000141749	0.000139895	0.000105811	0.000132629	0.000127248	0.000311948	0.000121727
IL12B	UNDETERMINED	UNDETERMINED	0.000360692	9.10815E-05	7.62536E-05	8.47888E-05	0.000187386	0.000158813
IL13	0.00022021	8.35642E-05	0.000306635	0.000114148	0.000212337	0.000195102	0.000221541	0.000149197
IL15	0.148103641	0.016978559	0.144019341	0.051065039	0.078566571	0.02733992	0.140509584	0.042597128
IL16	0.00012901	3.35257E-05	0.000259054	0.000316426	0.000198625	7.87302E-05	0.000193725	7.42609E-05

IL17A	0.000151286	2.6467E-05	UNDETERMINED	UNDETERMINED	7.78488E-05	0.00011193	0.000250374	0.000173233
IL17F	UNDETERMINED	UNDETERMINED	UNDETERMINED	UNDETERMINED	UNDETERMINED	UNDETERMINED	UNDETERMINED	UNDETERMINED
IL18	0.096956578	0.007606106	0.078956514	0.017809201	0.056883631	0.027142015	0.080561702	0.023771435
IL1A	0.000914331	0.000144605	0.197125453	0.057664998	0.086624528	0.011502342	0.001146309	0.000514798
IL1B	1.17583E-05	2.67684E-06	0.117457004	0.030440251	0.016442539	0.003418634	0.000242838	1.92906E-05
IL1RN	0.177337661	0.022139684	0.490456871	0.28785689	0.387602169	0.013091796	0.200886669	0.078247762
IL2	UNDETERMINED	UNDETERMINED	UNDETERMINED	UNDETERMINED	UNDETERMINED	UNDETERMINED	UNDETERMINED	UNDETERMINED
IL21	0.000373204	8.10196E-05	9.66892E-05	6.19414E-05	0.000193632	9.43324E-05	0.00029038	5.18026E-05
IL22	0.000112298	7.16409E-05	5.22906E-05	1.94391E-05	3.79554E-05	3.6524E-05	8.78349E-05	3.49475E-05
IL23A	0.002648016	0.000771173	0.005788964	0.002637733	0.002614074	0.000469311	0.002614478	0.000540522
IL24	0.000494582	4.74128E-05	0.00076398	0.000453082	0.000391162	9.91076E-05	0.000476279	5.46355E-05
IL27	UNDETERMINED	UNDETERMINED	UNDETERMINED	UNDETERMINED	UNDETERMINED	UNDETERMINED	UNDETERMINED	UNDETERMINED
IL3	0.00035958	0.000548828	9.69097E-05	UNDETERMINED	4.57836E-05	5.80214E-05	0.000109715	0.000120149
IL4	0.000524075	0.000131203	0.000652765	0.00029879	0.000336045	0.000163735	0.00060861	0.000298294
IL5	UNDETERMINED	UNDETERMINED	UNDETERMINED	UNDETERMINED	UNDETERMINED	UNDETERMINED	UNDETERMINED	UNDETERMINED
IL6	0.37363339	0.08490379	1.851550418	0.309345962	0.766841572	0.133091859	0.34846942	0.043343937
IL7	0.008886315	0.000737292	0.013076671	0.004697066	0.008412909	0.001916686	0.00944493	0.002179211
IL9	UNDETERMINED	UNDETERMINED	UNDETERMINED	UNDETERMINED	UNDETERMINED	UNDETERMINED	1.02059E-05	5.06546E-06
LIF	0.106672276	0.018332374	0.34146307	0.231746208	0.218749497	0.054441604	0.122133696	0.027070869
LOC517108	UNDETERMINED	UNDETERMINED	UNDETERMINED	UNDETERMINED	UNDETERMINED	UNDETERMINED	UNDETERMINED	UNDETERMINED
LTA	0.001250388	0.000165566	0.000230941	0.000164893	0.000424985	0.000111665	0.000437147	0.000207951
LTB	0.000667302	0.000337528	0.066844413	0.011851955	0.021062847	0.009008729	0.002222579	0.000501231
MIF	0.124256646	0.036822639	0.126032292	0.083507871	0.097660531	0.043769202	0.151854626	0.044475355
MSTN	0.000729557	0.000397207	0.000429704	0.00034269	UNDETERMINED	UNDETERMINED	0.001479854	0.001089591
NODAL	0.000275412	0.000202322	0.000186672	0.000206523	0.00021101	0.000171889	0.000428247	0.000270075
OSM	8.12657E-05	8.8655E-06	0.000177492	8.79929E-05	0.000125627	0.000135387	0.000204299	0.000142194
PF4	9.1248E-05	7.49133E-05	6.84874E-05	6.65168E-05	3.65133E-05	2.06592E-05	9.15677E-05	1.94175E-05
PPBP	2.46521E-05	2.12786E-05	0.000100436	0.000103311	UNDETERMINED	UNDETERMINED	0.000182535	0.000160436
SPP1	50.0174112	2.851806359	63.09440206	21.58729812	41.92038232	3.70725187	44.30464473	13.15111502
TGFB2	0.611999991	0.065897144	0.555345681	0.23028603	0.479969082	0.033645303	0.631735427	0.238326247
THPO	0.023508631	0.006132742	0.014541657	0.000675994	0.008376159	0.001305056	0.015939407	0.003263138
TNF	6.36649E-05	2.18017E-05	0.005038743	0.001301087	0.001916563	0.000740444	0.000104006	7.57022E-05
TNFRSF11B	0.005808006	0.000299442	0.061577266	0.021831814	0.038870572	0.007364977	0.00818581	0.001906551
TNFSF10	0.031926896	0.001809783	0.067618909	0.022159776	0.034676068	0.001813887	0.038385103	0.01316291
TNFSF11	UNDETERMINED	UNDETERMINED	UNDETERMINED	UNDETERMINED	UNDETERMINED	UNDETERMINED	UNDETERMINED	UNDETERMINED
TNFSF13B	0.002620171	0.000515001	0.004135004	0.002333424	0.001950745	0.001035621	0.002527487	0.001266248
VEGFA	0.514664887	0.024237691	0.557071508	0.172143047	0.286602316	0.111791869	0.473413243	0.093112602
XCL1	UNDETERMINED	UNDETERMINED	UNDETERMINED	UNDETERMINED	UNDETERMINED	UNDETERMINED	UNDETERMINED	UNDETERMINED
