# Supplementary material for: Regulation of inflammatory mediator expression in bovine endometrial cells: effects of lipopolysaccharide, interleukin 1 beta, and tumor necrosis factor alpha
Source: Physiol Rep. 2018 Apr 30;6(9):e13676. doi: 10.14814/phy2.13676 (PMC5925570; doi:10.14814/phy2.13676)
Supplement: Supplementary file 2 — Data S2. bCSC Gene expression (2−ΔΔCt). [file PHY2-6-e13676-s002.pdf]

bCSC Gene Expression (2^-ΔΔCt)

	Control		LPS		IL-1β		TNFα	
	Mean	SD	Mean	SD	Mean	SD	Mean	SD
ADIPOQ	UNDETERMINED	UNDETERMINED	UNDETERMINED	UNDETERMINED	UNDETERMINED	UNDETERMINED	UNDETERMINED	UNDETERMINED
BMP2	0.07159977	0.015572669	0.129769737	0.009517024	0.115353037	0.021892074	0.188116377	0.040904272
BMP3	UNDETERMINED	UNDETERMINED	0.001064606	0.00063733	0.001918563	0.00168744	UNDETERMINED	UNDETERMINED
BMP6	UNDETERMINED	UNDETERMINED	UNDETERMINED	UNDETERMINED	UNDETERMINED	UNDETERMINED	UNDETERMINED	UNDETERMINED
BMP7	0.001599136	0.000283916	0.003256648	0.000724653	0.004336386	0.001180847	0.002586842	8.86138E-05
C5	UNDETERMINED	UNDETERMINED	UNDETERMINED	UNDETERMINED	UNDETERMINED	UNDETERMINED	UNDETERMINED	UNDETERMINED
CCL1	0.000191691	4.84535E-05	0.000156351	5.47453E-05	0.000125957	2.36858E-05	0.000269387	8.40054E-05
CCL11	0.000323471	0.000231014	0.001719457	0.000330338	0.001858242	0.000345221	0.001258881	0.000144575
CCL17	0.00235684	0.001224991	0.00234608	0.000760833	0.001151	0.000208328	0.006127787	0.001998139
CCL19	UNDETERMINED	UNDETERMINED	0.000158874	5.50865E-05	UNDETERMINED	UNDETERMINED	0.000296522	0.000137174
CCL2	0.159823608	0.13858453	2.202216375	1.104884625	1.414914332	0.59636548	2.815771427	1.462568055
CCL20	4.53058E-05	4.54002E-05	0.005757592	0.001704046	0.021822808	0.007899715	0.000252206	0.000110602
CCL21	UNDETERMINED	UNDETERMINED	UNDETERMINED	UNDETERMINED	UNDETERMINED	UNDETERMINED	UNDETERMINED	UNDETERMINED
CCL22	UNDETERMINED	UNDETERMINED	UNDETERMINED	UNDETERMINED	UNDETERMINED	UNDETERMINED	UNDETERMINED	UNDETERMINED
CCL24	UNDETERMINED	UNDETERMINED	0.000210493	0.000131724	0.00018953	0.000122883	UNDETERMINED	UNDETERMINED
CCL3	UNDETERMINED	UNDETERMINED	0.000111573	0.000135628	0.000115644	5.46581E-05	7.29535E-05	3.94532E-05
CCL4	UNDETERMINED	UNDETERMINED	UNDETERMINED	UNDETERMINED	UNDETERMINED	UNDETERMINED	UNDETERMINED	UNDETERMINED
CCL5	0.001471004	0.000414209	0.201392941	0.06382835	0.065345978	0.001634656	0.274082397	0.064081469
CCL8	UNDETERMINED	UNDETERMINED	UNDETERMINED	UNDETERMINED	UNDETERMINED	UNDETERMINED	UNDETERMINED	UNDETERMINED
CD40LG	UNDETERMINED	UNDETERMINED	UNDETERMINED	UNDETERMINED	UNDETERMINED	UNDETERMINED	UNDETERMINED	UNDETERMINED
CD70	0.003730108	0.001242742	0.003153502	0.001631549	0.001845338	0.001231029	0.004205627	0.001119415
CNTF	0.001792027	0.000847266	0.002183408	0.001692568	0.002053208	0.001208097	0.001453411	0.000916178
CSF1	0.296084583	0.155458409	0.551960501	0.043331425	0.322493485	0.062430285	0.573072746	0.159145056
CSF2	0.003532659	0.000669091	0.112156679	0.050507487	0.425903181	0.187965315	0.012595296	0.00742326
CSF3	5.9496E-05	3.81115E-05	0.001197291	0.000231163	0.047823276	0.00616862	7.80733E-05	1.65188E-05
CTF1	0.002591249	0.001317703	0.001368915	0.00060093	0.00109458	0.000627863	0.001181135	0.000409575
CX3CL1	0.04748838	0.019409883	0.403362643	0.012881712	0.1145377	0.003074709	0.764906354	0.126504155
CXCL10	0.002224791	0.000866552	0.000932425	0.000417497	0.000329633	0.000199821	0.002348714	0.000792023
CXCL11	UNDETERMINED	UNDETERMINED	UNDETERMINED	UNDETERMINED	UNDETERMINED	UNDETERMINED	UNDETERMINED	UNDETERMINED
CXCL12	0.60504464	0.032814988	0.262675931	0.042934364	0.152580001	0.054964871	0.44920209	0.033077728
CXCL13	UNDETERMINED	UNDETERMINED	UNDETERMINED	UNDETERMINED	2.36014E-05	1.49649E-05	UNDETERMINED	UNDETERMINED
CXCL16	0.79144016	0.420206935	1.426591144	0.214911725	1.21786846	0.209788272	2.05301276	0.508894966
CXCL3	0.072944158	0.04085421	1.24304147	0.437551215	3.568366111	2.17604346	0.171315068	0.059147133
CXCL5	0.882611029	0.613108896	28.92245216	3.832491406	68.26005433	24.39901586	3.762160246	1.397998957
CXCL8	0.005969007	0.005795479	0.548784118	0.245467631	0.964326296	0.028985091	0.040909294	0.038764767
CXCL9	0.001818322	0.00043731	9.98699E-05	7.21252E-05	8.24801E-05	8.62736E-05	0.000356152	0.000180498
FASLG	UNDETERMINED	UNDETERMINED	UNDETERMINED	UNDETERMINED	0.000329922	0.00010183	UNDETERMINED	UNDETERMINED
GPI	1.21505845	0.478412907	1.230939599	0.308103708	1.270720374	0.257986917	1.318174548	0.101504652
GRO1	0.025210158	0.014344684	1.009443073	0.296241208	2.230258618	0.27494958	0.128620669	0.082290731
IFNG	UNDETERMINED	UNDETERMINED	UNDETERMINED	UNDETERMINED	UNDETERMINED	UNDETERMINED	UNDETERMINED	UNDETERMINED
IL10	0.000645491	0.000398631	0.001166268	0.000364106	0.000773042	0.000113532	0.000698355	0.00060826
IL12A	0.002099207	0.000897554	0.003753147	0.000908323	0.002386552	0.000759418	0.003684184	0.001801186
IL12B	UNDETERMINED	UNDETERMINED	0.000117793	0.000106309	0.000104702	6.81305E-05	3.49285E-05	3.61195E-05
IL13	0.000103808	7.0055E-05	UNDETERMINED	UNDETERMINED	0.000277102	0.000230143	5.15304E-05	6.74884E-05
IL15	0.000736015	0.000312262	0.003164678	0.001239959	0.001381488	0.000464379	0.003584505	0.001436325
IL16	0.00462594	0.002641908	0.008793562	0.002008382	0.004960302	0.000532436	0.011315076	0.003876615

IL17A	5.20517E-05	4.11766E-05	0.00020842	0.000262221	0.000177362	0.000135362	0.000145072	0.000117039
IL17F	UNDETERMINED	UNDETERMINED	UNDETERMINED	UNDETERMINED	UNDETERMINED	UNDETERMINED	UNDETERMINED	UNDETERMINED
IL18	0.319519874	0.103290304	0.356469635	0.047607037	0.261809936	0.050746496	0.429021752	0.136227235
IL1A	0.044423	0.026807202	0.686440637	0.138551872	0.751919858	0.177831599	0.152712865	0.047236682
IL1B	0.000582917	0.000665742	0.034443683	0.011306397	0.132560602	0.032127722	0.002265229	0.000615763
IL1RN	0.018173926	0.002476092	0.011591002	0.002603977	0.005856222	0.002464811	0.01781259	0.003533168
IL2	UNDETERMINED	UNDETERMINED	UNDETERMINED	UNDETERMINED	UNDETERMINED	UNDETERMINED	UNDETERMINED	UNDETERMINED
IL21	0.000124952	9.72826E-05	0.000292325	0.000243996	0.000313154	0.000159559	0.000240106	4.9101E-05
IL22	6.44259E-05	3.96277E-05	0.000121014	0.000112353	0.000168187	0.000124054	5.08648E-05	2.85927E-05
IL23A	0.000932113	0.000421438	0.001723773	0.000680789	0.001796925	0.000704872	0.001508246	0.000902285
IL24	0.000906121	0.000539304	0.000638151	0.000623245	0.000495148	0.000199605	0.000754544	0.000388958
IL27	UNDETERMINED	UNDETERMINED	UNDETERMINED	UNDETERMINED	0.00016524	9.40688E-05	UNDETERMINED	UNDETERMINED
IL3	UNDETERMINED	UNDETERMINED	UNDETERMINED	UNDETERMINED	0.000120457	0.000182611	UNDETERMINED	UNDETERMINED
IL4	0.00025986	0.000102381	0.000117347	0.00014482	UNDETERMINED	UNDETERMINED	0.000228565	0.000208329
IL5	UNDETERMINED	UNDETERMINED	7.00874E-05	6.30736E-05	UNDETERMINED	UNDETERMINED	UNDETERMINED	UNDETERMINED
IL6	0.050588167	0.033578595	2.267629699	1.337255108	4.57367832	0.015734506	0.369530043	0.162412196
IL7	0.054585522	0.027552138	0.083752644	0.010126758	0.082342627	0.020390855	0.084180105	0.035914035
IL9	UNDETERMINED	UNDETERMINED	UNDETERMINED	UNDETERMINED	0.000109279	0.000150664	UNDETERMINED	UNDETERMINED
LIF	0.006783404	0.003758255	0.030558524	0.010770191	0.054623411	0.034921245	0.011397089	0.004648224
LOC517108	UNDETERMINED	UNDETERMINED	UNDETERMINED	UNDETERMINED	UNDETERMINED	UNDETERMINED	UNDETERMINED	UNDETERMINED
LTA	0.000861687	0.000257089	0.00092758	0.000894006	0.001135872	0.000551535	0.000707936	0.000168878
LTB	UNDETERMINED	UNDETERMINED	0.000143567	9.62655E-05	0.000127772	0.000103856	6.14218E-05	3.43795E-05
MIF	0.144620093	0.077115379	0.131582529	0.017126365	0.095355966	0.018885312	0.142545708	0.032011971
MSTN	UNDETERMINED	UNDETERMINED	UNDETERMINED	UNDETERMINED	UNDETERMINED	UNDETERMINED	UNDETERMINED	UNDETERMINED
NODAL	0.001366238	0.000987718	0.000576304	0.000168197	0.0007993	0.000463804	0.000700087	0.000222437
OSM	3.16445E-05	2.16131E-05	8.84683E-05	9.69567E-05	9.55974E-05	8.20589E-05	6.43653E-05	6.45168E-05
PF4	0.000687074	0.000467131	0.003655661	0.000392691	0.005593414	0.000881754	0.001609765	0.000752254
PPBP	0.000369523	0.00020619	0.000607259	0.000281573	0.001112187	0.000152559	0.000725076	0.000481641
SPP1	11.83599555	3.122066687	4.750056862	1.072134907	2.820963269	0.382888735	5.8098942	1.180753556
TGFB2	0.064925652	0.026131826	0.046985184	0.006641262	0.037888479	0.002456202	0.075389978	0.013155351
THPO	0.002939928	0.001027218	0.001350119	0.000755458	0.001630439	0.001077701	0.001482197	0.000588234
TNF	UNDETERMINED	UNDETERMINED	0.000163399	0.000105877	0.000165883	7.5545E-05	0.000109565	8.1299E-06
TNFRSF11B	0.000401035	0.00022531	0.00145332	0.001361755	0.002824673	0.000228651	0.001739229	0.000328927
TNFSF10	0.001098207	0.000519849	0.003462653	0.001043407	0.001864891	0.000892204	0.006155028	0.004130925
TNFSF11	UNDETERMINED	UNDETERMINED	0.000435586	0.000202541	0.000701179	0.000843919	UNDETERMINED	UNDETERMINED
TNFSF13B	0.00458552	0.001272677	0.01543581	0.003388639	0.023802243	0.011787531	0.010750837	0.004869283
VEGFA	0.457058254	0.188119776	0.691081529	0.250365035	0.839372395	0.174221148	0.871151502	0.104249011
XCL1	UNDETERMINED	UNDETERMINED	UNDETERMINED	UNDETERMINED	UNDETERMINED	UNDETERMINED	UNDETERMINED	UNDETERMINED
